# Effects of obesity on post-surgical recovery and functional outcomes in rotator cuff tear repair

**DOI:** 10.3389/fsurg.2026.1772964

**Published:** 2026-06-03

**Authors:** Wuren Hou, Anpeng Xu, Minou Xu, Jian Chen

**Affiliations:** 1 Department of Orthopaedics, Sir Run Run Shaw Hospital, Zhejiang University School of Medicine, Hangzhou, China; 2 Department of Orthopaedics, The Second People's Hospital of Linhai City, Linhai, China

**Keywords:** arthroscopic repair, obesity, pain, range of motion, rotator cuff tear

## Abstract

**Background:**

Rotator cuff tears (RCTs) are a common cause of shoulder pain and can severely affect patients' ability to perform daily activities. Comorbidities, such as obesity, are known to influence the outcomes of RCT repair, particularly in the context of arthroscopic surgery. However, the effect of obesity on postoperative outcomes following RCT repair has not been sufficiently explored, especially in patients undergoing minimally invasive procedures. This study aims to evaluate the impact of obesity on postoperative pain, range of motion, and functional recovery after arthroscopic rotator cuff repair.

**Methods:**

This retrospective cohort study included 50 patients who underwent arthroscopic RCT repair between January 1, 2022, and January 1, 2025. All patients were followed for a minimum of two years postoperatively. Data collected included factors such as fatty infiltration (Goutallier grade), tendon retraction (Patte grade), obesity (BMI ≥ 30), education level, and smoking status. The primary outcomes assessed were postoperative pain (Visual Analog Scale, VAS), range of motion (active forward flexion and external rotation), UCLA Shoulder Score, and ASES Score.

**Results:**

The average age of the study participants was 59.9 ± 7.4 years, with 28% of patients being female. A total of 42 patients (84%) had a full-thickness rotator cuff tear. The median time from diagnosis to surgery was 22.5 months (IQR: 17.3–29.8 months), with a median follow-up duration of 30.2 months (IQR: 26.3–34.1 months). Eighteen patients (36%) were classified as obese (BMI ≥ 30). Obese patients demonstrated a trend toward higher postoperative pain (VAS: 4.8 ± 2.8 vs. 4.0 ± 3.2, *p* = 0.076), and showed significantly reduced active forward flexion (130.5° ± 48.4° vs. 137.6° ± 52.7°, *p* = 0.035) and external rotation (40.3° ± 17.4° vs. 48.4° ± 19.5°, *p* = 0.024) compared to non-obese patients. BMI was negatively correlated with improvements in both forward flexion and abduction following surgery.

**Conclusion:**

This study found that obesity was significantly associated with reduced active forward flexion (*p* = 0.035) and external rotation (*p* = 0.024) following arthroscopic rotator cuff repair, and demonstrated a trend toward higher postoperative pain levels (*p* = 0.076) that did not reach conventional statistical significance. Functional recovery, as assessed by UCLA and ASES scores, showed a consistent directional trend toward poorer outcomes in obese patients; however, these differences were not statistically significant (*p* = 0.196 and *p* = 0.322, respectively), and should be interpreted with caution given the limited sample size. These findings highlight obesity as a potential risk factor for reduced range of motion recovery following rotator cuff repair, while acknowledging that its impact on broader functional outcomes remains to be confirmed in larger prospective studies. Tailored perioperative and rehabilitation strategies targeting obese patients may help optimize postoperative recovery, though further evidence is needed to guide specific clinical recommendations.

## Introduction

Rotator cuff tears (RCTs) are among the most common causes of shoulder pain, particularly in middle-aged and elderly individuals (1). With aging, the rotator cuff tendons undergo degeneration, which increases the likelihood of tears (2). These tears may result from overuse, trauma, or age-related wear and tear. RCTs significantly impair shoulder function, limiting the ability to perform essential activities such as lifting, reaching overhead, or even basic tasks like combing hair or dressing. Surgical repair, particularly arthroscopic surgery, is the primary treatment for patients with rotator cuff tears, especially when conservative approaches like physical therapy and corticosteroid injections fail (3). Arthroscopic rotator cuff repair utilizes minimally invasive techniques, enabling surgeons to reattach torn tendons to the bone through small incisions with specialized instruments (4). This approach has proven effective in restoring shoulder function, reducing pain, and improving mobility for many patients. Compared to traditional open surgery, arthroscopic repair offers several advantages, including shorter recovery times, less postoperative pain, and a reduced risk of complications, such as infection or scarring (5). However, while arthroscopic rotator cuff repair has shown significant success in improving shoulder function, patients often face challenges during recovery. Complications such as retear of the rotator cuff or shoulder joint stiffness can impede recovery. Factors such as age, tendon quality, comorbidities, and adherence to rehabilitation protocols contribute to the variability of outcomes (6, 7), making it crucial for clinicians to consider individual patient factors when planning and managing postoperative care.

Obesity, defined as having a body mass index (BMI) of 30 or higher (8), is a growing global health issue. Particularly in developed countries, obesity rates are on the rise. Beyond being associated with general health problems such as cardiovascular disease and diabetes, obesity is also a significant risk factor for complications in orthopedic surgeries (9). Obese individuals typically experience delayed healing after surgery and a higher incidence of complications, including infection, wound dehiscence, and retear of repaired tissues. Additionally, obesity increases the mechanical load on weight-bearing joints, which may exacerbate pain and hinder recovery.

While the negative impact of obesity on outcomes in orthopedic surgeries is well-documented, its specific effects on rotator cuff repair, particularly in patients undergoing arthroscopic procedures, remain insufficiently studied. This study aims to assess the impact of obesity on postoperative outcomes following arthroscopic rotator cuff repair, focusing on pain, range of motion, and functional recovery. The primary outcomes being evaluated include postoperative pain, measured by the Visual Analog Scale (VAS), range of motion (including active forward flexion and external rotation), and functional recovery, assessed using the UCLA Shoulder Score and the ASES Score. The findings from this study could provide valuable insights for clinical decision-making, helping healthcare providers tailor interventions and optimize recovery strategies for obese patients undergoing rotator cuff repair, ultimately improving their long-term quality of life.

## Methods

### Study design and population

This study is a retrospective cohort study designed to evaluate the impact of obesity on postoperative outcomes following arthroscopic rotator cuff repair. The study aims to investigate how obesity affects key recovery parameters such as pain, range of motion, and functional recovery after surgery, providing insights into the challenges faced by obese patients undergoing rotator cuff repair. The study population consists of patients who underwent arthroscopic rotator cuff repair at the hospital between January 1, 2022, and January 1, 2025. Inclusion criteria include patients who had a confirmed diagnosis of rotator cuff tear and underwent arthroscopic surgery during the specified timeframe. To ensure the validity of the study results, patients must have had complete preoperative and postoperative data available, including imaging, clinical assessments, and follow-up evaluations for at least two years after surgery. Exclusion criteria include patients with incomplete follow-up data, prior shoulder surgeries, or other significant musculoskeletal disorders that could confound the results. Such disorders include shoulder fractures or severe rotator cuff muscle degeneration that could impact the healing process and recovery outcomes. Additionally, patients with conditions that may significantly affect healing, such as active cancer, uncontrolled systemic diseases, or other comorbidities that could impair the rehabilitation process, be excluded from the study.

### Data collection

Active forward flexion and external rotation were measured using a standard handheld goniometer by trained orthopaedic clinical staff at each scheduled follow-up visit. Measurements were performed with the patient in a standardized seated position following a standardized protocol. Assessors were not blinded to patient BMI classification, which represents a potential source of measurement bias. Inter-observer variability was not formally assessed in this study, which is acknowledged as a limitation.

Data have been collected on various demographic and clinical characteristics of the patients prior to their arthroscopic rotator cuff repair. These variables included age, sex, body mass index (BMI), smoking history, education level, and relevant comorbidities. BMI has been calculated for each patient and classified into three categories: normal weight (BMI < 25), overweight (BMI 25–29.9), and obese (BMI ≥ 30), based on standard BMI thresholds. Smoking history has been categorized as current smoker, former smoker, or non-smoker. Education level was divided into two categories: ≤8 years of formal education and >8 years of formal education. Education level was recorded as a potential proxy for socioeconomic status and health literacy, both of which may influence rehabilitation compliance and postoperative outcomes. Due to sample size constraints, education level was not included as a covariate in the primary regression models but is reported descriptively in Table 1. Comorbidities such as diabetes, hypertension, cardiovascular disease, and other relevant health conditions have been recorded as present or absent for each patient.

**Table 1:** Patient characteristics.

**Characteristic**	**Value**
Male	36 (72%)
Female	14 (28%)
Age (mean ± SD)	59.9 ± 7.4
RCT Cause	
Degenerative RCT Lesion	38 (76%)
Trauma	12 (24%)
Full-thickness tear	42 (84%)
Dominant side operated	35 (70%)
Occupation	
Sedentary	40 (80%)
Manual labor	10 (20%)
Arthroscopic repair	50 (100%)
Smokers	32 (64%)
Literacy	
≤ 8 years	40 (80%)
> 8 years	10 (20%)
BMI	
Normal	20 (40%)
Overweight	12 (24%)
Obese	18 (36%)
Comorbidities	
Cardiovascular disease	5 (10%)
Diabetes mellitus	13 (26%)
Dyslipidemia	22 (44%)
Hypertension	26 (52%)
Hand osteoarthritis	15 (30%)
Knee osteoarthritis	18 (36%)
Previous shoulder corticosteroid infiltration	16 (32%)

Data represent mean (SD) and N (%) of 50 patients subjected to RCT repair. BMI, body mass index; RCT, rotator cuff tear.

Imaging data have been collected prior to surgery to assess the severity of the rotator cuff tear. This included x-ray and MRI images to determine whether the tear was partial or full-thickness, and to evaluate any associated tendon retraction or fatty infiltration. The Goutallier classification has been used to assess the degree of fatty infiltration in the rotator cuff muscles, ranging from grade 0 (no fatty infiltration) to grade 4 (severe fatty infiltration). The Patte classification has been employed to evaluate tendon retraction, ranging from grade 1 (mild retraction) to grade 4 (severe retraction) (10).

All patients have been followed for a minimum of two years after their rotator cuff repair surgery. During this period, clinical assessments have been conducted at regular intervals to monitor recovery. Key postoperative measures have included pain levels, range of motion (ROM), and functional recovery. Pain has been assessed using the Visual Analog Scale (VAS), where 0 represents no pain and 10 represents the worst possible pain (11). Active forward flexion and external rotation have been measured to assess the recovery of shoulder mobility (12). Functional recovery has been evaluated using the UCLA Shoulder Score and the ASES (American Shoulder and Elbow Surgeons) Score, which assess pain, function, and overall shoulder health (13). Additionally, any complications such as infection, retear of the rotator cuff, or adverse events during recovery have been recorded throughout the follow-up period.

### Postoperative rehabilitation protocol

All patients followed a standardized postoperative rehabilitation protocol supervised by experienced physiotherapists. The protocol consisted of three phases: (1) an immobilization phase (weeks 0–6) involving sling immobilization with pendulum exercises to minimize joint stiffness while protecting the repaired tendon; (2) an active-assisted range of motion phase (weeks 6–12) incorporating progressive passive and active-assisted shoulder exercises aimed at gradually restoring shoulder mobility; and (3) a strengthening phase (weeks 12–24) comprising progressive resistance exercises targeting the rotator cuff musculature and periscapular stabilizers to restore functional shoulder strength. Physical therapy sessions were conducted twice weekly at the outpatient rehabilitation department throughout the rehabilitation period. Compliance with the rehabilitation protocol was monitored and documented at each scheduled follow-up visit. Patients who missed more than two consecutive sessions were contacted and the reasons for non-attendance were recorded.

### Outcome measures

Pain level has been assessed using the Visual Analog Scale (VAS), a widely used measure of pain intensity. The VAS ranges from 0 to 10, where 0 represents no pain and 10 represents the worst possible pain.

ROM has been assessed through measurements of active forward flexion and external rotation (ER) of the affected shoulder. These two movements are key indicators of shoulder mobility and function. Active forward flexion measures the ability to lift the arm forward, while external rotation assesses the ability to rotate the arm outward from the shoulder.

Functional recovery has been evaluated using two commonly used shoulder outcome scores: the UCLA Shoulder Score and the ASES (American Shoulder and Elbow Surgeons) Score. The UCLA Shoulder Score evaluates pain, function, and overall shoulder condition, while the ASES Score focuses on the severity of shoulder symptoms and the patient's ability to perform daily activities.

### Statistical analysis

For the primary analysis, patients were dichotomized into two groups based on BMI: obese (BMI ≥ 30 kg/m^2^) and non-obese (BMI < 30 kg/m^2^). A three-category BMI classification (normal weight: BMI < 25.0 kg/m^2^; overweight: BMI 25.0–29.9 kg/m^2^; obese: BMI ≥ 30 kg/m^2^) was used exclusively for descriptive characterization of the study population, as presented in Table 1.

Baseline characteristics, including age, sex, BMI, smoking history, education level, and comorbidities (e.g., diabetes, hypertension, and cardiovascular disease), were summarized using means and standard deviations (SD) for continuous variables and frequencies with percentages for categorical variables. Baseline comparability between the obese and non-obese groups was assessed using independent samples t-tests for continuous variables, where normality assumptions were satisfied, and Mann–Whitney U tests where they were not. Chi-square tests were applied for categorical variables. All comparisons are reported with 95% confidence intervals (CIs) and corresponding *p*-values.

Prior to all inferential analyses, the normality of continuous outcome variables was formally assessed within each subgroup using the Shapiro–Wilk test, which is recommended for small sample sizes. For variables satisfying normality assumptions (Shapiro–Wilk *p* > 0.05), between-group comparisons of postoperative outcomes — including pain (VAS), range of motion (ROM), and functional recovery (UCLA and ASES scores) — were performed using independent samples t-tests. Where normality assumptions were violated (Shapiro–Wilk *p* ≤ 0.05), non-parametric Mann–Whitney U tests were applied as alternatives for two-group comparisons, and Kruskal–Wallis tests were used for comparisons involving all three BMI categories. All between-group comparisons are reported with mean differences or median differences as appropriate, accompanied by 95% CIs and corresponding *p*-values to facilitate transparent interpretation of effect magnitude and precision.

To assess the magnitude of recovery from preoperative baseline within each group, change scores were calculated for all ROM and functional outcomes as the difference between postoperative and preoperative values (*Δ* = postoperative−preoperative). To account for potential residual baseline differences between BMI groups, analysis of covariance (ANCOVA) was subsequently performed, incorporating preoperative ROM and functional scores as covariates. ANCOVA-adjusted *p*-values are reported alongside unadjusted comparisons to allow direct assessment of the robustness of the observed associations independent of baseline status.

The associations between BMI and improvements in ROM and functional recovery scores were evaluated using Pearson's correlation coefficient for normally distributed variables and Spearman's rank correlation coefficient for non-normally distributed variables, as determined by the Shapiro–Wilk test. It is important to note that these correlation analyses represent univariable, unadjusted assessments of the linear association between BMI and outcome improvements, and should be interpreted distinctly from the adjusted multivariable regression effects reported in Table 2.

**Table 2:** Multiple regression analysis.

**Outcome**	**Predictor**	**B**	**95% CI**	***p*-value**	**Adjusted R^2^**
VAS	BMI	0.14	0.02, 0.26	0.023	0.18
	Age	0.03	−0.05, 0.11	0.431	
	Sex	−0.21	−1.12, 0.70	0.645	
	Goutallier grade	0.18	−0.04, 0.40	0.107	
Active FF (°)	BMI	−1.23	−2.31, −0.15	0.027	0.21
	Age	−0.42	−1.08, 0.24	0.208	
	Sex	3.14	−8.62, 14.90	0.596	
	Goutallier grade	−2.31	−5.18, 0.56	0.113	
Active ER (°)	BMI	−0.87	−1.64, −0.10	0.029	0.19
	Age	−0.18	−0.52, 0.16	0.291	
	Sex	1.24	−4.38, 6.86	0.661	
	Goutallier grade	−1.02	−2.48, 0.44	0.168	
UCLA Score	BMI	−0.31	−0.89, 0.27	0.289	0.09
	Age	−0.12	−0.38, 0.14	0.354	
	Sex	0.86	−3.24, 4.96	0.678	
	Goutallier grade	−0.54	−1.68, 0.60	0.348	
ASES Score	BMI	−1.12	−3.45, 1.21	0.341	0.07
	Age	−0.43	−1.52, 0.66	0.435	
	Sex	2.18	−14.32, 18.68	0.793	
	Goutallier grade	−2.86	−7.54, 1.82	0.228	

B: unstandardized regression coefficient. Each model was adjusted for age, sex, and Goutallier grade as covariates. The number of covariates was restricted to four per model to minimize overfitting risk given the sample size of *n* = 50. ASES, American Shoulder and Elbow Surgeons Shoulder Score; BMI, body mass index; CI, confidence interval; ER, external rotation; FF, forward flexion; UCLA, University of California at Los Angeles Shoulder Score; VAS, visual analog scale.

Multiple linear regression models were constructed to evaluate the independent association between BMI and each postoperative outcome after adjustment for potential confounders. Given the sample size of 50 patients, the number of covariates per model was restricted to a maximum of four, in accordance with the widely accepted guideline of approximately one predictor per ten subjects, to minimize the risk of model overfitting and instability. Covariates were selected *a priori* based on established clinical relevance: age, sex, and Goutallier grade were included alongside BMI as the primary predictor of interest. Patte grade was considered for inclusion but was ultimately excluded due to sample size constraints and its non-significant univariable association with outcomes in this cohort (Table 3), as simultaneously incorporating five predictors would have exceeded the recommended covariate limit. Model fit was assessed using the adjusted R^2^ statistic. To avoid overinterpretation, results from multivariable regression models — reported as unstandardized regression coefficients (B) with 95% CIs and *p*-values (Table 2) — are interpreted as reflecting independent associations between BMI and outcomes after covariate adjustment, and are explicitly distinguished from the unadjusted correlation coefficients presented in the correlation analyses above.

**Table 3:** Impact of fatty infiltration and tendon retraction in RCT repair.

	UCLA shoulder score (mean ± SD)	VAS pain score (mean ± SD)	ASES score (mean ± SD)
Patte Classification			
Patte 1	27.8 (7.2)	3.5 (3.2)	60.3 (38.7)
Patte 2	26.5 (7.1)	3.9 (2.8)	56.5 (32.4)
Patte 3	25.3 (6.2)	5.6 (3.3)	45.6 (31.6)
*P*-value	0.711	0.216	0.333
Goutallier Grade			
Goutallier 0–2	28.4 (9.2)	2.3 (3.2)	70.0 (37.1)
Goutallier 3–4	25.6 (7.3)	4.6 (3.1)	49.3 (32.6)
*P*-value	0.173	0.088	0.106

50 patients underwent rotator cuff tear (RCT) surgical repair. Data represent mean (SD). ASES, American Shoulder and Elbow Surgeons score; BMI, body mass index; ER, external rotation; FF, forward flexion; UCLA, University of California, Los Angeles Shoulder score; VAS, visual analog scale. All reported outcome values represent postoperative assessments at final follow-up.

Throughout all analyses, association-based wording is used consistently to describe the relationship between BMI and postoperative outcomes (e.g., “BMI was associated with,” “higher BMI showed a significant association with”). Causal language has been deliberately avoided given the observational, retrospective nature of this study, which precludes causal inference.

The correlation between BMI and improvements in ROM was assessed using Pearson's correlation coefficient for normally distributed variables and Spearman's rank correlation coefficient for non-normally distributed variables, as determined by the Shapiro–Wilk test.

All statistical analyses were performed using SPSS version 26.0 (IBM Corp., Armonk, NY, USA). A two-tailed *p*-value of less than 0.05 was considered statistically significant. Given the exploratory nature of this study and the limited sample size, no formal correction for multiple comparisons was applied; all findings should therefore be interpreted with appropriate caution as hypothesis-generating rather than confirmatory.

### Ethical considerations

This study was conducted in full accordance with the ethical principles outlined in the Declaration of Helsinki and all applicable institutional and national regulations governing human subjects research. Ethical approval was obtained from the Institutional Review Board (IRB) of Sir Run Run Shaw Hospital, Zhejiang University School of Medicine, prior to the initiation of any study-related activities (Approval No. S2022008). Any subsequent amendments to the study protocol were submitted to the IRB for review and approval before implementation. Written informed consent was obtained from all participants prior to their enrollment in the study. All patients were fully informed of the study's purpose, procedures, potential risks and benefits, and the intended use of their personal medical data. Strict confidentiality was maintained throughout the study, and all patient data were anonymized and handled in accordance with applicable data protection regulations. Participation was entirely voluntary, and patients retained the right to withdraw from the study at any time without consequence to their medical care or treatment.

## Result

### Study population

The study included a total of 50 patients who underwent arthroscopic RCT repair. The cohort consisted of 36 male patients (72%) and 14 female patients (28%), with an average age of 59.9 ± 7.4 years. The majority of patients had a full-thickness rotator cuff tear (84%), with 38 patients (76%) presenting with a degenerative lesion and 12 patients (24%) experiencing trauma as the underlying cause. The dominant shoulder was operated on in 35 patients (70%). A significant proportion of the study population led a sedentary lifestyle, with 32 patients (64%) reporting minimal physical activity. All patients (100%) underwent arthroscopic repair. In terms of smoking status, 40% of patients were smokers, and 80% of the cohort had received ≤8 years of formal education. Regarding body mass index (BMI), 36% of patients were classified as obese (BMI ≥ 30), 24% as overweight (BMI 25–29.9), and 40% as having a normal weight (BMI < 25). In terms of comorbidities, 52% had hypertension, 44% had dyslipidemia, 26% had diabetes mellitus, 10% had cardiovascular disease, and 36% had a history of previous shoulder corticosteroid infiltration. These clinical and demographic characteristics are summarized in Table 1.

### Impact of fatty infiltration and tendon retraction in RCT repair

Table 3 summarizes the effects of fatty infiltration (Goutallier grade) and tendon retraction (Patte grade) on postoperative outcomes in patients who underwent rotator cuff tear (RCT) repair. The data show that higher grades of fatty infiltration and tendon retraction were associated with poorer functional recovery and increased pain levels. Specifically, patients with tendon retraction graded as Patte 3 experienced lower UCLA scores (25.3 ± 6.2) compared to those with Patte 1 (27.8 ± 7.2) and Patte 2 (26.5 ± 7.1), though the differences were not statistically significant (*P* = 0.711). Similarly, patients with higher Goutallier grades (3–4) showed worse functional recovery, with a mean ASES score of 49.3 ± 32.6 compared to 70.0 ± 37.1 in those with Goutallier grades 0–2, but this difference also did not reach statistical significance (*P* = 0.106). In terms of pain, the VAS scores did not show significant differences between the groups, with a mean of 3.5 ± 3.2 for Patte 1, 3.9 ± 2.8 for Patte 2, and 5.6 ± 3.3 for Patte 3 (*P* = 0.216). However, higher fatty infiltration (Goutallier 3–4) did show a trend toward more pain (VAS: 4.6 ± 3.1) compared to Goutallier 0–2 (VAS: 2.3 ± 3.2), but this difference was also not statistically significant (*P* = 0.088). These findings suggest that while fatty infiltration and tendon retraction may influence functional recovery and pain levels, their impact on postoperative outcomes is not significantly different in this cohort.

### Impact of obesity in RCT repair

Table 4 summarizes the preoperative baseline values, postoperative outcomes, and change scores for all clinical measures, stratified by BMI group (BMI ≥ 30 kg/m^2^ vs. BMI < 30 kg/m^2^).

**Table 4:** Impact of BMI on surgical outcomes.

Outcome	Time Point	BMI ≥ 30 kg/m^2^ (*n* = 18)	BMI < 30 kg/m^2^ (*n* = 32)	Mean Difference (95% CI)	*p*-value
VAS	Preoperative	6.8 ± 2.1	6.5 ± 2.3	0.3 (−0.9, 1.5)	0.621
	Postoperative	4.8 ± 2.8	4.0 ± 3.2	0.8 (−0.1, 1.7)	0.076
	Change Score (*Δ*)	−2.0 ± 1.8	−2.5 ± 2.0	0.5 (−0.6, 1.6)	0.347
	ANCOVA-adjusted p	—	—	—	0.081
Active FF (°)	Preoperative	98.2 ± 32.1	101.4 ± 29.8	−3.2 (−19.8, 13.4)	0.703
	Postoperative	130.5 ± 48.4	137.6 ± 52.7	−7.1 (−13.6, −0.6)	0.035
	Change Score (*Δ*)	+32.3 ± 28.6	+36.2 ± 31.4	−3.9 (−19.2, 11.4)	0.041
	ANCOVA-adjusted p	—	—	—	0.038
Active ER (°)	Preoperative	28.6 ± 12.3	30.1 ± 11.8	−1.5 (−8.4, 5.4)	0.661
	Postoperative	40.3 ± 17.4	48.4 ± 19.5	−8.1 (−15.2, −1.0)	0.024
	Change Score (*Δ*)	+11.7 ± 10.2	+18.3 ± 12.6	−6.6 (−13.1, −0.1)	0.028
	ANCOVA-adjusted p	—	—	—	0.026
UCLA Score	Preoperative	14.2 ± 5.1	15.1 ± 4.8	−0.9 (−3.7, 1.9)	0.521
	Postoperative	25.5 ± 7.6	27.5 ± 6.6	−2.0 (−5.1, 1.1)	0.196
	Change Score (*Δ*)	+11.3 ± 6.8	+12.4 ± 5.9	−1.1 (−4.8, 2.6)	0.551
	ANCOVA-adjusted p	—	—	—	0.204
ASES Score	Preoperative	32.4 ± 18.6	34.8 ± 17.2	−2.4 (−13.2, 8.4)	0.651
	Postoperative	57.5 ± 28.6	62.6 ± 30.8	−5.1 (−16.8, 6.6)	0.322
	Change Score (*Δ*)	+25.1 ± 22.3	+27.8 ± 24.1	−2.7 (−15.8, 10.4)	0.681
	ANCOVA-adjusted p	—	—	—	0.334

Data represent mean ± SD. Change Score (*Δ*) = postoperative value minus preoperative value. ANCOVA was performed with preoperative values included as covariates to adjust for baseline differences between groups. ASES, American Shoulder and Elbow Surgeons Shoulder Score; ANCOVA, analysis of covariance; BMI, body mass index; CI, confidence interval; ER, external rotation; FF, forward flexion; UCLA, University of California at Los Angeles Shoulder Score; VAS, visual analog scale.

Preoperative baseline values were comparable between the two BMI groups across all outcome measures, with no statistically significant differences observed in preoperative VAS scores (6.8 ± 2.1 vs. 6.5 ± 2.3, *p* = 0.621), active forward flexion (98.2° ± 32.1° vs. 101.4° ± 29.8°, *p* = 0.703), external rotation (28.6° ± 12.3° vs. 30.1° ± 11.8°, *p* = 0.661), UCLA scores (14.2 ± 5.1 vs. 15.1 ± 4.8, *p* = 0.521), or ASES scores (32.4 ± 18.6 vs. 34.8 ± 17.2, *p* = 0.651) between obese and non-obese patients, respectively. These findings indicate that the two groups were well-matched at baseline, reducing the likelihood of baseline confounding in subsequent comparisons.

At final follow-up, obese patients demonstrated a trend toward higher postoperative pain levels, with a mean VAS score of 4.8 ± 2.8 compared to 4.0 ± 3.2 in the non-obese group; however, this difference did not reach conventional statistical significance (mean difference: 0.8, 95% CI: −0.1–1.7, *p* = 0.076). Analysis of change scores similarly showed no significant between-group difference in pain reduction (*Δ*: −2.0 ± 1.8 vs. −2.5 ± 2.0, *p* = 0.347), suggesting that while obese patients tended to report higher absolute pain levels, the magnitude of pain improvement following surgery was comparable between groups.

Obese patients demonstrated significantly reduced postoperative range of motion compared to non-obese patients in both active forward flexion (130.5° ± 48.4° vs. 137.6° ± 52.7°, mean difference: −7.1°, 95% CI: −13.6 to −0.6, *p* = 0.035) and external rotation (40.3° ± 17.4° vs. 48.4° ± 19.5°, mean difference: −8.1°, 95% CI: −15.2 to −1.0, *p* = 0.024). Consistent with these findings, analysis of change scores confirmed that obese patients achieved significantly smaller improvements in active forward flexion (*Δ*: +32.3° ± 28.6° vs. + 36.2° ± 31.4°, *p* = 0.041) and external rotation (*Δ*: +11.7° ± 10.2° vs. + 18.3° ± 12.6°, *p* = 0.028) from preoperative baseline compared to their non-obese counterparts. These associations remained statistically significant after ANCOVA adjustment for preoperative baseline ROM values (forward flexion: *p* = 0.038; external rotation: *p* = 0.026), confirming the robustness of these observations independent of preoperative status.

With respect to functional outcomes, the UCLA Shoulder Score for obese patients was 25.5 ± 7.6, marginally lower than the 27.5 ± 6.6 recorded in non-obese patients, though this difference did not reach statistical significance (mean difference: −2.0, 95% CI: −5.1 to 1.1, *p* = 0.196). Similarly, the ASES score for obese patients (57.5 ± 28.6) was lower than that of non-obese patients (62.6 ± 30.8), but this difference also failed to achieve statistical significance (mean difference: −5.1, 95% CI: −16.8 to 6.6, *p* = 0.322). Change score analyses were consistent with these findings, with no significant between-group differences in UCLA improvement (*Δ*: +11.3 ± 6.8 vs. + 12.4 ± 5.9, *p* = 0.551) or ASES improvement (*Δ*: +25.1 ± 22.3 vs. + 27.8 ± 24.1, *p* = 0.681). ANCOVA-adjusted analyses incorporating preoperative functional scores as covariates yielded comparable results (UCLA: *p* = 0.204; ASES: *p* = 0.334).

Taken together, these findings indicate that obesity was significantly associated with reduced postoperative range of motion in both active forward flexion and external rotation, with these associations confirmed by both change score analyses and ANCOVA-adjusted models. A non-significant trend toward higher postoperative pain was also observed in obese patients. In contrast, the impact of obesity on broader functional recovery, as assessed by the UCLA and ASES scores, did not reach statistical significance in this exploratory cohort, although a consistent directional trend toward poorer outcomes in obese patients was noted across all measures. Given the limited sample size, these functional trends warrant further investigation in larger prospective studies.

**Figure 1:**
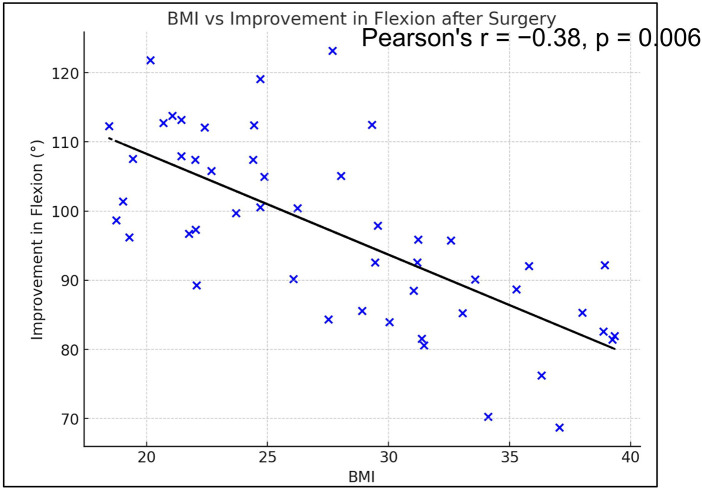
Scatter plot illustrating the correlation between body mass index (BMI) and improvement in active forward flexion following arthroscopic rotator cuff repair. Pearson's r = −0.38, *p* = 0.006. Each data point represents one patient (*n* = 50). The regression line with 95% confidence interval is shown.

Figure 1 shows the correlation between the patient's BMI and the improvement in shoulder forward flexion after rotator cuff tear (RCT) repair, as assessed by Pearson's correlation coefficient (r). The data reveals a negative correlation between BMI and the improvement in forward flexion, suggesting that higher BMI values are associated with less improvement in the range of motion following surgery. As BMI increases, the improvement in forward flexion decreases, with patients in the higher BMI categories showing smaller gains in flexion compared to those with lower BMI. This correlation is statistically significant, emphasizing the influence of obesity on postoperative shoulder mobility.

**Figure 2:**
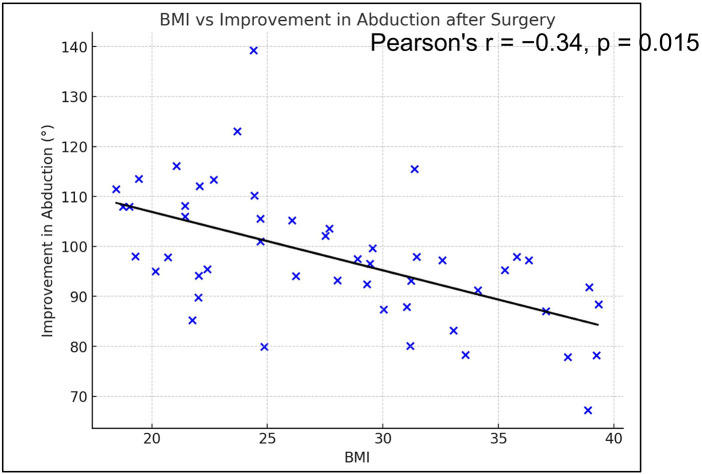
Scatter plot illustrating the correlation between body mass index (BMI) and improvement in shoulder abduction following arthroscopic rotator cuff repair. Pearson's r = −0.34, *p* = 0.015. Each data point represents one patient (*n* = 50). The regression line with 95% confidence interval is shown.

Figure 2 illustrates a similar correlation, this time between BMI and the improvement in shoulder abduction after surgery. As with forward flexion, there is a negative correlation between BMI and improvement in abduction. Patients with a higher BMI had less improvement in shoulder abduction following surgery compared to those with a lower BMI. The trend seen in Figure 2 mirrors the findings in Figure 1, highlighting that obesity may limit the recovery of shoulder mobility, particularly in movements such as abduction. Both figures underscore the importance of considering BMI as a factor influencing recovery after arthroscopic rotator cuff repair.

### Postoperative complications

During the follow-up period, complications were recorded in a minority of patients. Two patients (4%) experienced superficial wound infections, both of which resolved with oral antibiotic therapy. Three patients (6%) were identified as having radiologically confirmed retear of the repaired rotator cuff on follow-up MRI, all of whom were in the obese group (BMI ≥ 30). No deep infections, neurovascular injuries, or other major surgical complications were recorded. Given the small number of complications, formal between-group statistical comparisons were not performe.

### Multivariable regression analysis of predictors of postoperative outcomes

To further evaluate the independent association between BMI and postoperative outcomes after adjusting for potential confounders, multiple linear regression analyses were performed for each outcome measure. Each model included BMI as the primary predictor, with age, sex, and Goutallier grade retained as covariates. Given the sample size of 50 patients, the number of covariates was restricted to a maximum of four per model to minimize the risk of overfitting. Full regression results are presented in Table 4.

With respect to postoperative pain, BMI was identified as a statistically significant independent predictor of VAS score (B = 0.14, 95% CI: 0.02–0.26, *p* = 0.023), indicating that each unit increase in BMI was associated with a 0.14-point increase in postoperative pain score after adjustment for age, sex, and Goutallier grade. The overall model accounted for 18% of the variance in postoperative VAS scores (adjusted R^2^ = 0.18).

Regarding range of motion, BMI was also a significant independent predictor of postoperative active forward flexion (B = −1.23, 95% CI: −2.31 to −0.15, *p* = 0.027), with each unit increase in BMI associated with a 1.23° reduction in forward flexion after covariate adjustment (adjusted R^2^ = 0.21). Similarly, BMI independently predicted postoperative external rotation (B = −0.87, 95% CI: −1.64 to −0.10, *p* = 0.029), with each unit increase in BMI associated with a 0.87° reduction in external rotation (adjusted R^2^ = 0.19). These findings are consistent with the between-group comparisons reported in Table 3 and reinforce the association between higher BMI and reduced postoperative shoulder mobility.

In contrast, BMI was not a statistically significant independent predictor of either UCLA Shoulder Score (B = −0.31, 95% CI: −0.89 to 0.27, *p* = 0.289; adjusted R^2^ = 0.09) or ASES Score (B = −1.12, 95% CI: −3.45 to 1.21, *p* = 0.341; adjusted R^2^ = 0.07) after adjustment for covariates. The relatively low adjusted R^2^ values for these functional outcome models suggest that BMI and the included covariates collectively explain only a modest proportion of the variance in functional recovery scores, consistent with the non-significant between-group differences observed in Table 3.

Among the covariates, Goutallier grade demonstrated a consistent directional trend toward poorer outcomes across all models, though it did not reach statistical significance in any individual model, likely reflecting limited statistical power. Age and sex did not emerge as significant independent predictors in any of the regression models.

Taken together, the multivariable regression analyses confirm that higher BMI is independently associated with greater postoperative pain and reduced range of motion following arthroscopic rotator cuff repair, even after adjustment for age, sex, and degree of fatty infiltration. However, BMI did not independently predict functional recovery as assessed by UCLA and ASES scores, suggesting that its impact on broader shoulder function may be attenuated by other unmeasured factors or may require larger samples to detect.

## Discussion

This study found that obesity significantly impacts postoperative outcomes following arthroscopic rotator cuff repair. Obese patients demonstrated significantly reduced range of motion in both active forward flexion (*p* = 0.035) and external rotation (*p* = 0.024), and showed a trend toward higher postoperative pain (*p* = 0.076) and poorer functional recovery compared to non-obese patients. These findings suggest that obesity is a potential contributing factor associated with reduced range of motion recovery after rotator cuff surgery, while its impact on pain and functional scores warrants further investigation in larger studies.

### Interpretation of key findings

The trend toward higher VAS scores in obese patients (*p* = 0.076) suggests a possible association between obesity and increased postoperative pain following rotator cuff repair, though this difference did not reach conventional statistical significance. This may be attributed to several potential mechanisms. First, obesity is associated with a heightened inflammatory response, which could exacerbate pain and delay the healing process. Excess adipose tissue releases pro-inflammatory cytokines that could increase local inflammation around the surgical site, leading to prolonged pain (14). Additionally, the mechanical stress exerted by excess weight on the shoulder joint may increase strain on the repaired rotator cuff, contributing to discomfort and delayed recovery (15). This combination of factors may contribute to the trend toward elevated pain levels noted in the obese cohort.

Obese patients demonstrated a reduced range of motion, particularly in active forward flexion and external rotation, which may stem from both physical and metabolic factors. The excess body weight can alter biomechanics, leading to increased joint stress and restricted movement (16). Obesity may also impact tissue healing by impairing the vascular supply to the affected shoulder, potentially leading to slower recovery and stiffness (17). Furthermore, the increased load on the joint can contribute to mechanical limitations, making it more difficult for the shoulder to achieve full mobility. The combination of these factors likely results in the diminished range of motion observed in obese patients following surgery.

Although the differences in UCLA and ASES scores did not reach statistical significance, the trend toward lower scores in obese patients suggests that obesity may negatively influence functional recovery. Obesity is known to affect muscle regeneration and strength, which are essential for post-surgical rehabilitation. Muscle atrophy, often seen in obese individuals, can hinder the recovery of shoulder strength, resulting in less favorable functional outcomes (18). Additionally, the healing process may be delayed in obese patients, as they often have compromised tissue regeneration and altered response to rehabilitation. These factors, combined with the mechanical stresses placed on the shoulder joint, likely contribute to the poorer functional recovery observed in the obese cohort.

### Clinical implications

Given the significant impact of obesity on postoperative outcomes, it is crucial to consider obesity as a major risk factor when developing preoperative and postoperative care plans for rotator cuff repair patients. Obese patients may require more careful planning to ensure optimal recovery. Specific interventions should include weight management strategies to reduce the excess load on the shoulder joint and promote healing. This could involve nutritional counseling, weight loss programs, and regular monitoring of weight throughout the treatment and recovery process. Additionally, targeted rehabilitation protocols should be designed to accommodate the unique needs of obese patients. These protocols may involve more gradual progression of physical therapy and closer monitoring for potential complications, such as delayed wound healing or infection, that are more prevalent in obese patients.

Tailored rehabilitation programs are essential for addressing the unique challenges faced by obese patients, including limited mobility, slower recovery rates, and potential musculoskeletal imbalances. A one-size-fits-all approach to rehabilitation may not be effective for this population, and individualized plans are necessary to optimize recovery. Early intervention is critical to avoid prolonged periods of immobility, which can lead to further stiffness and weakness. Physical therapy programs should emphasize joint mobility, strength training, and stretching exercises aimed at improving range of motion and shoulder function. Incorporating advanced rehabilitation techniques, such as functional strength training, progressive resistance exercises, and manual therapy, could help enhance outcomes for obese patients by addressing both muscle atrophy and stiffness.

### Study strengths and limitations

#### Strengths of the study

This study offers several notable strengths that enhance the validity and clinical relevance of its findings. First, the study focuses on a well-defined patient population — individuals undergoing arthroscopic rotator cuff repair — which improves the internal consistency of the cohort and strengthens the applicability of findings to this specific surgical context. By systematically examining multiple BMI categories, the study provides a nuanced understanding of how varying degrees of adiposity may differentially influence postoperative recovery, thereby offering more granular insights to inform individualized patient management strategies.

Second, the retrospective cohort design, while inherently limited in certain respects, offers distinct practical advantages. It enables the examination of real-world clinical outcomes across a consecutive patient series without the constraints of prospective enrollment, reflecting the everyday complexities encountered in orthopaedic practice. The inclusion of comprehensive preoperative and postoperative data — encompassing imaging parameters, validated functional outcome scores, and standardized range of motion measurements — further strengthens the methodological rigor of the study within its design framework.

#### Limitations of the study

This study should be interpreted in light of several important limitations, which are discussed in order of methodological priority.

The most critical limitation is the relatively small sample size (*n* = 50), with only 18 patients classified as obese. This substantially restricts statistical power, particularly for multivariable regression analyses and subgroup comparisons. A *post-hoc* power analysis confirmed that the study was underpowered to detect moderate effect sizes (Cohen's d < 0.5) for several secondary outcomes, including UCLA and ASES scores. Accordingly, all findings should be regarded as exploratory and hypothesis-generating rather than confirmatory, and adequately powered prospective studies with larger cohorts are needed to validate these observations.

A closely related methodological concern pertains to residual confounding by tear morphology. Although Goutallier grade was incorporated as a covariate in all primary regression models to adjust for fatty infiltration severity, Patte grade could not be simultaneously included due to sample size constraints and the associated risk of model overfitting. Residual confounding by tendon retraction severity therefore cannot be entirely excluded. Future studies with larger sample sizes should aim to include both Goutallier and Patte grades as covariates in adjusted analyses to more comprehensively account for the influence of tear morphology on postoperative outcomes.

The retrospective design of this study introduces additional inherent limitations. Reliance on existing medical records increases the risk of incomplete or inconsistent data, which may affect the accuracy and completeness of the findings. Furthermore, the absence of randomization raises the possibility of selection bias, as patients with more severe obesity or greater comorbidity burden may have received different perioperative management compared to those with lower BMI, introducing systematic differences that cannot be fully accounted for through statistical adjustment alone.

The observational nature of this study also fundamentally precludes causal inference. The observed associations between obesity and postoperative outcomes may be influenced by unmeasured confounders, including physical activity levels, nutritional status, psychosocial factors, and individual variations in pain perception. Prospective randomized or controlled designs would be necessary to establish causal directionality, and all findings should accordingly be interpreted as associative rather than causal.

A further limitation relates to the duration of follow-up. Although a minimum two-year follow-up period provides meaningful information on short- to medium-term recovery trajectories, it does not fully capture the long-term impact of obesity on rotator cuff repair outcomes. Extended follow-up would be valuable to determine whether the observed associations between obesity and reduced range of motion persist, attenuate, or worsen over time, and whether the non-significant trends in functional recovery scores ultimately reach clinical or statistical significance with continued rehabilitation.

Finally, the study could not fully control for several additional sources of variability that may have influenced outcomes independently of BMI, including differences in surgical technique, surgeon experience, and individual adherence to postoperative rehabilitation protocols. Future studies should incorporate these variables as covariates or employ randomized controlled trial designs to more rigorously isolate the specific contribution of obesity to rotator cuff repair recovery.

## Conclusion

This study found that obesity was significantly associated with reduced active forward flexion (*p* = 0.035) and external rotation (*p* = 0.024) following arthroscopic rotator cuff repair, and demonstrated a trend toward higher postoperative pain levels (*p* = 0.076) that did not reach conventional statistical significance. Functional recovery, as assessed by UCLA and ASES scores, showed a trend toward poorer outcomes in obese patients; however, these differences were not statistically significant (*p* = 0.196 and *p* = 0.322, respectively), and should be interpreted with caution given the limited sample size. These findings highlight obesity as a potential risk factor for reduced range of motion recovery following rotator cuff repair, while acknowledging that its impact on broader functional outcomes remains to be confirmed in larger prospective studies. Tailored perioperative and rehabilitation strategies targeting obese patients may help optimize postoperative recovery, though further evidence is needed to guide specific clinical recommendations.

## Data Availability

The original contributions presented in the study are included in the article/Supplementary Material, further inquiries can be directed to the corresponding author.

## References

[1] Liao H Yu HP Song W Zhang G Lu B Zhu YJ. Amorphous calcium phosphate nanoparticles using adenosine triphosphate as an organic phosphorus source for promoting tendon-bone healing. J Nanobiotechnology. (2021) 19:270. 10.1186/s12951-021-01007-y 34493293 PMC8425074

[2] Wang Z Long Z Li H Lu H Gingery A Amadio PC. A biomechanical comparison of a mesh suture to a polyblend suture in a porcine tendon model. Ann Transl Med. (2021) 9:450. 10.21037/atm-20-1065 33850847 PMC8039690

[3] Kara YS Hapa O Işın Y Kılıç Aİ Havitçioğlu H. A comparison of ice wrap and subacromial injection for postoperative pain and edema control following arthroscopic rotator cuff repair. J Orthop Traumatol. (2020) 21:17. 10.1186/s10195-020-00556-6 32876791 PMC7468014

[4] Routledge JC Saber AY Pennington N Gupta N. Re-Tear rates following rotator cuff repair surgery. Cureus. (2023) 15:e34426. 10.7759/cureus.34426 36874651 PMC9981227

[5] Liu Z Lu H Yuan Y Fu Z Xu H. Mid-term follow-up evaluation of a new arthroscopic broström procedure for chronic lateral ankle instability. J Orthop Surg Res. (2023) 18:316. 10.1186/s13018-023-03789-3 37095551 PMC10123977

[6] Alsubheen SA MacDermid JC John FK James OT. Factors predicting postoperative range of motion and muscle strength one year after shoulder arthroplasty. Arch Bone Jt Surg. (2021) 9:399–405. 10.22038/abjs.2020.48521.2405 34423087 PMC8359654

[7] Ozbaydar MU Tonbul M Tekin AC Yalaman O. Arthroscopic rotator cuff repair: evaluation of outcomes and analysis of prognostic factors. Acta Orthop Traumatol Turc. (2007) 41:169–74. PMID: 17876114 17876114

[8] Petrou NA Rafique H Rasheed S Tekkis P Kontovounisios C. Colorectal cancer and the obese patient: a call for guidelines. Cancers (Basel). (2022) 14(21):5255. 10.3390/cancers14215255 36358674 PMC9657704

[9] McMullan M Millar R Woodside JV. A systematic review to assess the effectiveness of technology-based interventions to address obesity in children. BMC Pediatr. (2020) 20:242. 10.1186/s12887-020-02081-1 32438908 PMC7243328

[10] Pihl E Skorpil M Sköldenberg O Hedbeck CJ Jonsson KB. At mid- to long-term follow-up after proximal hamstring tendon avulsion; there was greater fatty infiltration, muscle atrophy and strength deficit in the hamstring muscles of the injured leg than in the uninjured leg. J Orthop Surg Res. (2023) 18:114. 10.1186/s13018-023-03582-2 36797740 PMC9933258

[11] Zhao P Cui Y Sun L Sun X. Inhalation of low-dose desflurane prevents the hemodynamic instability caused by target-controlled infusion of remifentanil and propofol during laparoscopic gynecological surgery: a randomized controlled trial. Exp Ther Med. (2021) 21:54. 10.3892/etm.2020.9486 33273982 PMC7706382

[12] Ho S Denard PJ Chong XL Collin P Wang S Lädermann A. Achilles tendon-bone block allograft for massive rotator cuff tears with bony deficiency of the greater tuberosity: a Minimum 2-year follow-up study. Orthop J Sports Med. (2022) 10:941678775. 10.1177/23259671211073719 PMC8873559 35224116

[13] Kim J Nam JH Kim Y Kim JS Kim SH. Long head of the Biceps tendon tenotomy versus subpectoral tenodesis in rotator cuff repair. Clin Orthop Surg. (2020) 12:371–78. 10.4055/cios19168 32904028 PMC7449864

[14] Abshirini M Coad J Wolber FM von Hurst P Miller MR Tian HS. Effects of Greenshell™ mussel intervention on biomarkers of cartilage metabolism, inflammatory markers and joint symptoms in overweight/obese postmenopausal women: a randomized, double-blind, and placebo-controlled trial. Front Med (Lausanne). (2022) 9:1063336. 10.3389/fmed.2022.1063336 36544504 PMC9760926

[15] Davidson PA Rivenburgh DW. Rotator cuff repair tension as a determinant of functional outcome. J Shoulder Elbow Surg. (2000) 9:502–06. 10.1067/mse.2000.109385 11155303

[16] Allen WE Lin JJ Barfield WB Friedman RJ Eichinger JK. Shoulder motion decreases as body mass increases in patients with asymptomatic shoulders. JSES Int. (2020) 4:438–42. 10.1016/j.jseint.2020.04.004 32939465 PMC7479022

[17] Martino D Gulotta I V L. The effect of obesity in shoulder arthroplasty outcomes and complications. Orthop Clin North Am. (2018) 49:353–60. 10.1016/j.ocl.2018.02.010 29929717

[18] Wang H Peng H Zhang L Gao W Ye J. Novel insight into the relationship between muscle-fat and bone in type 2 diabetes ranging from normal weight to obesity. Diabetes Metab Syndr Obes. (2022) 15:1473–84. 10.2147/DMSO.S364112 35586203 PMC9109979

